# Inflammatory pseudotumor around metal-on-polyethylene total hip arthroplasty in patients with ankylosing spondylitis: description of two cases and review of literature

**DOI:** 10.1186/s12957-015-0487-8

**Published:** 2015-02-15

**Authors:** Dong Fu, Wei Sun, Jiakang Shen, Xiaojun Ma, Zhengdong Cai, Yingqi Hua

**Affiliations:** Department of Orthopaedics, Shanghai First People’s Hospital, School of Medicine, Shanghai Jiaotong University, Shanghai, 200080 China

**Keywords:** Inflammatory pseudotumor, Metal-on-polyethylene, THR, Ankylosing spondylitis

## Abstract

Inflammatory pseudotumor has been commonly reported in patients undertaking total hip replacement (THR) for different reasons. The precise etiology of this biological reaction and whether the primary disease has an influence on pseudotumor formation remain unclear. There seems to be a consensus that metal ions and debris do play an important role during this process. Recently, however, compared to metal particles along, immune response induced by metal particles attracts more attention. We present two cases of pseudotumor who have accepted THR for ankylosing spondylitis (AS) and later required revision surgery and hindquarter amputation, respectively. By thorough literature review, we tried to investigate the association between inflammatory pseudotumors and immunology.

## Background

Since 1974, when periarticular mass was first described by Evans *et al*. [1] in one case undertaking the first-generation metal-on-metal (MOM) hip replacement, it has long been recognized as a new type of complication occurring around the hip prosthesis. According to the record of revised cases, the incidence of pseudotumors reached 1.8%, and it increased in patients who received bilateral hip replacement [2-4]. For total hip replacement (THR), it was estimated that 1% of patients would develop pseudotumors 5 years postoperatively, and the incidence seemed to keep growing with a longer follow-up [5]. However, the exact etiology of such soft tissue reactions remains a poorly understood event. Later published studies have reported periprosthetic masses with different terms as inflammatory pseudotumors [6], bursae [7], cysts [8], or adverse reactions to metal debris [9]. Most previous studies observed a link between MOM articulation and pseudotumors [10-12]. Interestingly, pseudotumors associated with osteolysis and periprosthetic bone loss are now being increasingly reported in patients with polyethylene-on-metal (POM) hip replacement [2,13-15]. Pseudotumors causing severe symptoms including pain, neuropathy, bone loss, and loosening of prosthetic components always required revision surgery or amputation in a majority of patients [2]. The reported outcome of revision surgery with pseudotumors during follow-up was much more inferior compared to the outcome of non-pseudotumors prosthesis revisions [16]. It brought us a question about how pseudotumors influenced the artificial hip joint and surrounding structures and what were the potential risk factors associated with the development of these masses.

It remains controversial whether pseudotumors are mediated by primarily wear debris-induced granulomatous reaction or an immune response. There are various theories in the literature with respect to pseudotumor formation. Two cases who have undertaken THR for ankylosing spondylitis presented with a large soft tissue mass around a failed prosthesis were present in this study. Written informed consent was obtained from the patient. We hypothesize that except the previous reported mechanisms, pseudotumor was partly developed due to the immune response related to ankylosing spondylitis (AS) in our cases.

## Case presentation

### Case 1

**Figure 1:**
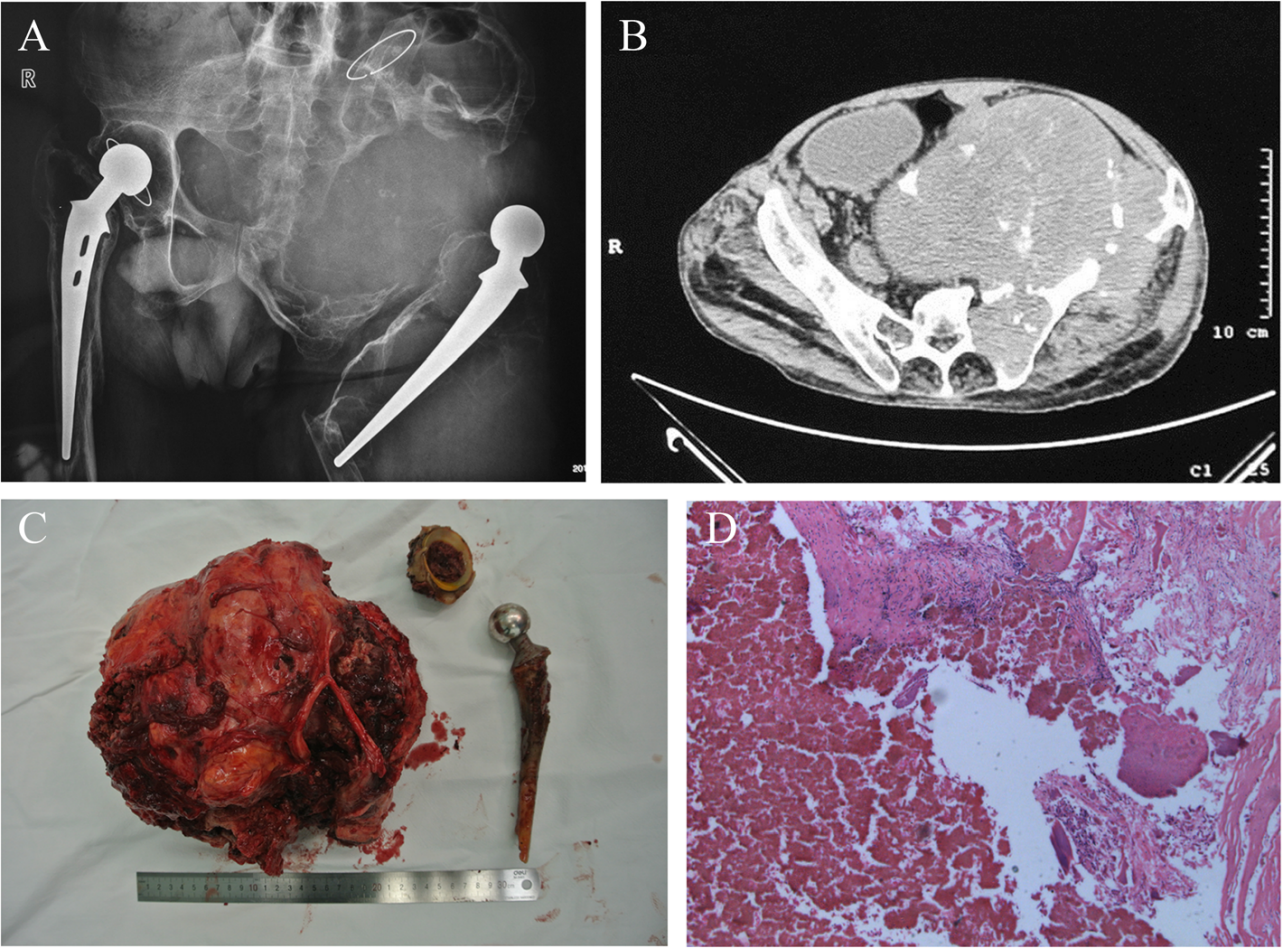
**A 60-year-old man with ankylosing spondylitis. (A)** X film showed a massive periprosthetic osteolytic lesion associated with huge soft tissue mass around a failed total hip replacement that has taken up the left periumbilical region. Pathological fracture occurred at the distal part of femoral stem. **(B)** CT scan confirmed huge soft tissue component in the pelvis. **(C)** We performed a hemipelvic amputation and non-significant polyethylene wear was detected. **(D)** Histology afterward revealed no sarcoma, but inflammatory tissue.

A 60-year-old man had bilateral total hip replacement 20 years ago due to ankylosing spondylitis. In the recent 5 years, he found increasing swelling in the left pelvis and a mass in the left thigh. The patient was referred to different hospitals with no definitive diagnosis, because he received one fine needle aspiration (FNA) biopsy without malignant histology evidence. On June 2013, he was admitted to our sarcoma center because pathological fracture occurred. X film showed a massive periprosthetic osteolytic lesion associated with huge soft tissue mass around a failed total hip replacement that has taken up the left periumbilical region. Computed tomographic (CT) scan confirmed huge soft tissue component in the pelvis. We also found one fistula on the skin of the mass. He had a low fever, and blood test showed an elevated white cell count (11.8 × 109/L). C-reactive protein increased to 75.3 mg/L, and erythrocyte sedimentation rate also raised to 24 mm/h. Based on his long history of arthroplasty and radiographic findings, clinical diagnosis of implant-induced sarcoma or chronic inflammatory mass was made. We gave him another FNA biopsy showing no tumor cells, but mainly necrosis. Considering that low chance of preserving a functional limb, we performed a hemipelvic amputation. Histology afterward revealed no sarcoma, but inflammatory tissue. The patient accomplished stage one wound healing and was discharged with no other complications (Figure 1).

### Case 2

**Figure 2:**
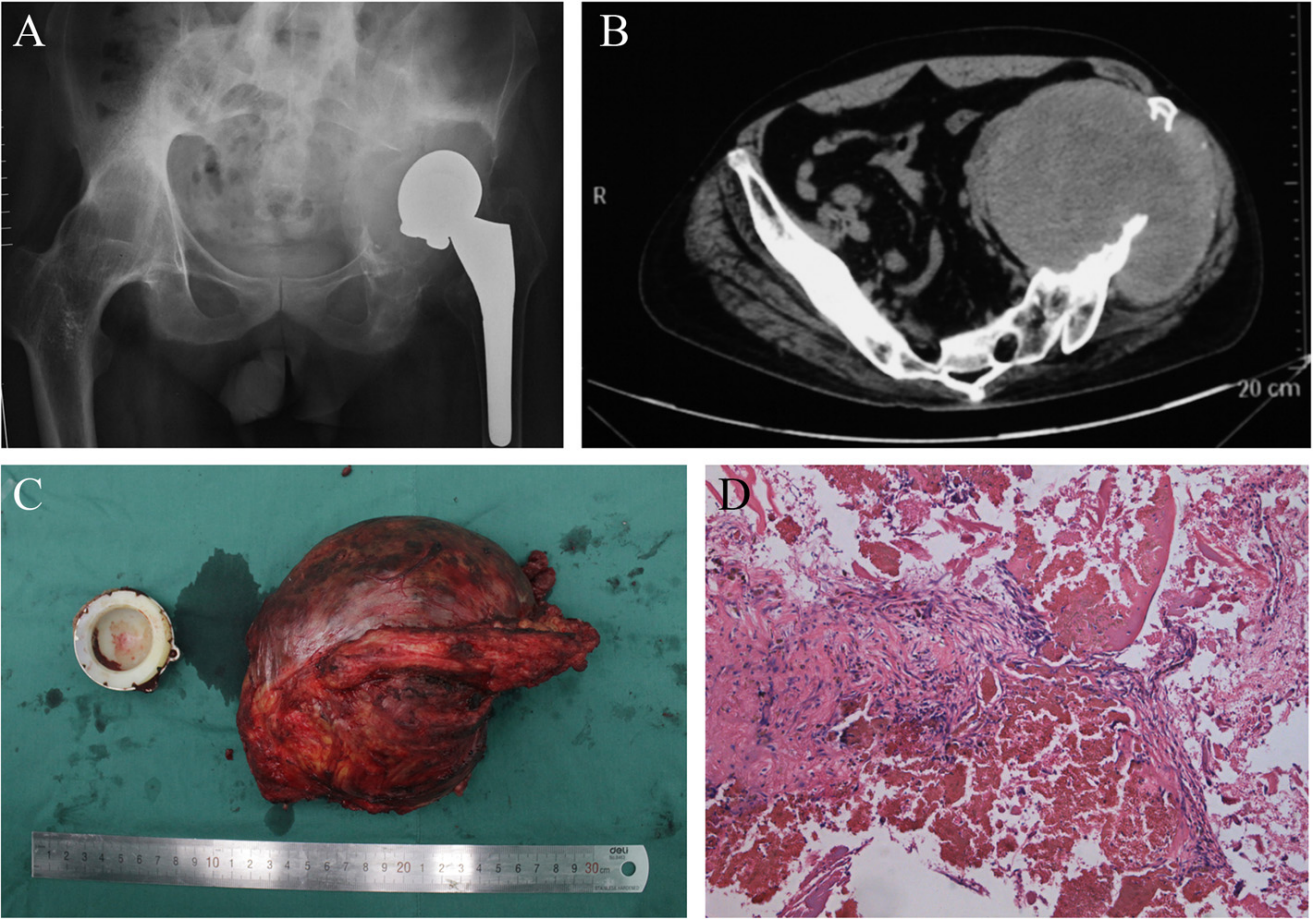
**A 47-year-old man with ankylosing spondylitis. (A)** X film showed a large soft tissue around the dislocated THA extending to pelvic. **(B)** Computed tomographic (CT) scan at admission showed a huge mass extending to the pelvic with serious bone destruction. **(C)** We performed hemipelvic reconstruction, and non-significant polyethylene wear was detected. **(D)** Postoperative histopathology showed proliferation of fibrovascular tissue and fibroadipose tissue around the canal and the adjacent soft tissue along with a few scattered lymphocytes. No malignant lesions were detected but mainly a lot of necrosis.

On May 2014, a 47-year-old man was admitted to our hospital complaining of progressive pain, swelling, and disability of the left hip during the past 6 years. He was diagnosed with ankylosing spondylitis for more than 30 years and received an uncemented total hip arthroplasty in 1986. In 2008, he noticed a feeling of mild to moderate pain of the ipsilateral hip but did not seek medical attention at that time. Over the following years, the pain continued to be tolerant associated with some new emerging symptoms (swelling and disability of the left hip). An incidental finding on a film taken in 2014 due to an accidental fall showed a large soft tissue around the dislocated THA extending to pelvic. On physical examination, an obvious and tender lump was palpated around the left hip. No signs of infection were observed. CT scan at admission showed huge mass extending to the pelvic with serious bone destruction. Laboratory analysis of inflammatory markers showed slightly elevated white cell count at 11.4 × 109/L, C-reactive protein at 8.10 mg/L, and erythrocyte sedimentation rate at 30 mm/h. In the present case, revision of the total acetabular and femoral component by hemipelvic reconstruction was necessary given the patient’s age, previous limb function, mass size, and bone destruction. The result of FNA before surgery ruled out infection and malignant tumors. Postoperative histopathology showed proliferation of fibrovascular tissue and fibroadipose tissue around the canal and the adjacent soft tissue along with a few scattered lymphocytes. No malignant lesions were detected but mainly a lot of necrosis. The surgery and early postoperative rehabilitation were uncomplicated (Figure 2).

### Discussion

To the best of our knowledge, this study for the first time described inflammatory pseudotumors in AS patients after THR. Since pseudotumor is commonly reported and so well known, one joint surgeon may ignore the differential diagnosis which may lead to the development of periprosthetic soft tissue mass [17]. Such pathologic changes could be induced by a number of other factors like metabolic disease, infection, and even neoplasia. Recently, however, the potential relationship between periprosthetic mass and musculoskeletal neoplasms has been reported. Considering the serious consequences, differential diagnosis of tumors should be kept in mind despite the relative lower incidence. Once misdiagnosed, one patient will miss the optimal treatment options and suffer an irreparable disaster. Fabbri *et al*. previously provided one case who had to accept hindquarter amputation and ultimately died because of a failed diagnosis of angiosarcoma [17]. Similar case reports of angiosarcoma have also been published from several other authors [18,19]. In addition, cases of osteogenic sarcoma, malignant fibrous histiocytoma, and epithelioid sarcoma were also reported from different centers with an average follow-up from 4 to 10 years after implant insertion [20-23]. In animal models, Carter *et al*. has successfully induced sarcomas in rats with solid and fragmented polyethylene [24]. Therefore, the presence of a tumor needs to be included in the differential diagnosis, and more attention should be paid considering that THR is widely used in both young and elder patients. It was difficult to distinguish pseudotumors from a necrotic tumor based on the imageological or pathological examination along. Detailed review of clinical courses, discreet physical examination, careful evaluation of standard X-ray, CT, MRI, and histological examinations of biopsy specimens should be performed in order to increase diagnostic accuracy.

Several authors have suggested that pseudotumors are a reaction to high wear [2,25] while other studies provided evidence showing that pseudotumor formation depends more on metal hypersensitivity [26,27]. The conception at this time is that pseudotumors are an adverse reaction which consists of a spectrum of inflammatory responses to metal debris. Whether this is immunologically mediated or a unique response to particles, or both, and which factor plays the dominant role are still unclear. In addition, the causes for immune induction and subsequent immune effect remain controversial [28].

De Smet *et al*. [29] previously proved that serum metal ion concentrations had a positive correlation with the degree of metal wear. There are many studies supporting the point that higher serum metal ion concentrations will be inevitably detected in patients with pseudotumors compared to those without [25]. Young-Min *et al*. showed elevated levels of cobalt and chromium ions in seven patients with pseudotumors after MOM hip resurfacing arthroplasty.

The term aseptic lymphocyte-dominated vasculitis associated lesion (ALVAL) which consists of the examination of synovial lining integrity, perivascular lymphocytes infiltrate, and tissue organization has been used to promote more standardized reporting of histopathologic changes of hip surrounding tissues [30]. Among those specific characteristics, perivascular lymphocytes attributing to a systemic reaction have been especially referred to in numerous studies [30-33].

Langton *et al*. [9] have compared results of histological examination of 17 patients who accepted revision surgery for an adverse response to metal debris and those revised for other reasons. They found significantly higher component wear and blood ion levels in the former patients. However, the result of ALVAL showed a lower level of lymphocytic infiltrates in patients with high metal wear. The dominant macroscopic feature was metallosis in patients with highly increased metal ion levels, while those with moderate metal levels showed tissue necrosis which meant a linkhood between lower metal debris but high metal sensitivity and tissue necrosis. The presence of high levels of lymphocytic infiltrates in patients with low wear is consistent with our observations. Caicedo *et al*. [34] has always emphasized that immune reactivity to debris should be the primary cause of inflammatory pseudotumors. He demonstrated a relationship between the thickness of perivascular lymphocytic cuffs and surface layer necrosis and then got a conclusion consistent with Langton that tissue necrosis seems more likely to be the consequence of an immune response induced by the metal debris rather than the metal toxicity.

Campbell *et al*. [35] conducted a study to compare the histological difference of pseudotumor in two populations (patients in group 1 were suspected of having high wear and patients in group 2 were suspected of having metal hypersensitivity). Using a 10-point histological scale of ALVAL, they found tissues from group 1 had a lower ALVAL score, fewer perivascular lymphocytes infiltrate, but were associated with more metal particles compared to those from group 2. It was likely that compared to high metal wear, metal hypersensitivity played a more important role in pseudotumor-like reactions. Higher hypersensitivity with lower wear also induced significant immune response in patients after THR.

The role of inflammatory cells in inflammasome pathway has been deeply investigated by Caicedo *et al*. who opened a new sight into the pathway of debris-induced inflammation [34]. They have tested effects of Co2^+^, Cr3^+^, Mo5^+^, Ni2^+^, and cobalt alloy particles on both upstream and downstream components of the inflammasome pathway in human macrophages with different concentrations. The results showed that all the tested ions and particles activated both upstream and downstream components of inflammasome pathways in a concentration-dependent model. In addition, a further investigation of intracellular molecular mechanisms of the inflammasome pathway was carried. This time, Caicedo showed metal ions and particles could activate caspase-1 which subsequently promotes the transformation and secretion of IL-b from pro-IL-1b. IL-b has been recognized as one important inductor for broader proinflammatory response. It was able to feedback and activate NFkb pathway, leading to a production of other proinflammatory cytokines (for example, IL-6 and TNFa).

Except the local effects of implants debris, we must also consider the systemic effects that can provoke this immune response. Ankylosing spondylitis is a systemic rheumatic disease, meaning it affects the entire system. The relationship between AS and HLA-B27 indicates the condition involving CD8 T cells, which interact with HLA-B27. In addition, HLA-B27 seems to be a unique protein associated with a number of special characteristics among which interacting with T cell receptors in association with CD4 shows extensive possibility. What is more, observation from previous studies showed that tumor necrosis factor-alpha (TNF α) and IL-1 are implicated in ankylosing spondylitis [36]. It is therefore, possible that immune response associated with AS may have also been responsible for metal hypersensitivity and, hence, pseudotumor formation. In our cases, non-significant polyethylene wear was detected at the time of revision surgery which may be attributed to the fact that being restricted by previous function level, patients who undergo THR as a result of AS are prone to have a lower physical activity which result in a lower incidence of polyethylene wear.

## Conclusions

In conclusion, we have described the occurrence of typical pseudotumors in two AS patients 20 and 28 years after primary THR, respectively. Both blood tests and histopathologic examination indicate a relative strong immune response to metal debris. Even though we have no clear evidence, it is quite possible that the development of pseudotumors in AS patients was not coincidental. Compared with previous studies, the majority of findings point to a significant link between immune response and pseudotumor formation. In addition, prosthesis or implant-related sarcoma is very rare, but should be recognized as a long-term complication after arthroplasty. Surgeons need to be aware of tumor as a differential diagnosis during clinical diagnosis of patients with inflammatory pseudotumor. Furthermore, basic studies focusing on investigating the mechanism by which pseudotumors and tumors are induced after THR ought to deserve more attention.

## Consent

Written informed consent was obtained from the patient for publication of this case report and any accompanying images. A copy of the written consent is available for review by the Editor-in-Chief of this journal.

## Abbreviations

THR: total hip replacement
AS: ankylosing spondylitis

## References

[1] Evans EM, Freeman MA, Miller AJ, Vernon-Roberts B (1974). Metal sensitivity as a cause of bone necrosis and loosening of the prosthesis in total joint replacement. J Bone Joint Surg Br.

[2] Pandit H, Glyn-Jones S, McLardy-Smith P, Gundle R, Whitwell D, Gibbons CL (2008). Pseudotumours associated with metal-on-metal hip resurfacings. J Bone Joint Surg.

[3] Hart AJ, Sabah S, Henckel J, Lewis A, Cobb J, Sampson B (2009). The painful metal-on-metal hip resurfacing. J Bone Joint Surg.

[4] Glyn-Jones S, Pandit H, Kwon YM, Doll H, Gill HS, Murray DW (2009). Risk factors for inflammatory pseudotumour formation following hip resurfacing. J Bone Joint Surg.

[5] Mabilleau G, Kwon YM, Pandit H, Murray DW, Sabokbar A (2008). Metal-on-metal hip resurfacing arthroplasty: a review of periprosthetic biological reactions. Acta Orthop.

[6] Boardman DR, Middleton FR, Kavanagh TG (2006). A benign psoas mass following metal-on-metal resurfacing of the hip. J Bone Joint Surg.

[7] De Smet KA (2005). Belgium experience with metal-on-metal surface arthroplasty. Orthop Clin North Am.

[8] Gruber FW, Bock A, Trattnig S, Lintner F, Ritschl P (2007). Cystic lesion of the groin due to metallosis: a rare long-term complication of metal-on-metal total hip arthroplasty. J Arthroplasty.

[9] Langton DJ, Jameson SS, Joyce TJ, Hallab NJ, Natu S, Nargol AV (2010). Early failure of metal-on-metal bearings in hip resurfacing and large-diameter total hip replacement: a consequence of excess wear. J Bone Joint Surg.

[10] Jasty MJ, Floyd WE, Schiller AL, Goldring SR, Harris WH (1986). Localized osteolysis in stable, non-septic total hip replacement. J Bone Joint Surg Am.

[11] Maloney WJ, Jasty M, Harris WH, Galante JO, Callaghan JJ (1990). Endosteal erosion in association with stable uncemented femoral components. J Bone Joint Surg Am.

[12] Schmalzried TP, Jasty M, Harris WH (1992). Periprosthetic bone loss in total hip arthroplasty. Polyethylene wear debris and the concept of the effective joint space. J Bone Joint Surg Am.

[13] Wang JW, Lin CC (1996). Pelvic mass caused by polyethylene wear after uncemented total hip arthroplasty. J Arthroplasty.

[14] Leigh W, O’Grady P, Lawson EM, Hung NA, Theis JC, Matheson J (2008). Pelvic pseudotumor: an unusual presentation of an extra-articular granuloma in a well-fixed total hip arthroplasty. J Arthroplasty.

[15] Clayton RA, Beggs I, Salter DM, Grant MH, Patton JT, Porter DE (1988). Inflammatory pseudotumor associated with femoral nerve palsy following metal-on-metal resurfacing of the hip. A case report. J Bone Joint Surg Am.

[16] Grammatopolous G, Pandit H, Kwon YM, Gundle R, McLardy-Smith P, Beard DJ (2009). Hip resurfacings revised for inflammatory pseudotumour have a poor outcome. J Bone Joint Surg.

[17] Fabbri N, Rustemi E, Masetti C, Kreshak J, Gambarotti M, Vanel D (2011). Severe osteolysis and soft tissue mass around total hip arthroplasty: description of four cases and review of the literature with respect to clinico-radiographic and pathologic differential diagnosis. Eur J Radiol.

[18] McDonald DJ, Enneking WF, Sundaram M (2002). Metal-associated angiosarcoma of bone: report of two cases and review of the literature. Clin Orthop Relat Res.

[19] Vives P, Sevestre H, Grodet H, Marie F (1987). Malignant fibrous histiocytoma of the femur after total hip prosthesis. Apropos of a case. Rev Chir Orthop Reparatrice Appar Mot.

[20] Swann M (1984). Malignant soft-tissue tumour at the site of a total hip replacement. J Bone Joint Surg.

[21] Penman HG, Ring PA (1984). Osteosarcoma in association with total hip replacement. J Bone Joint Surg.

[22] Bago-Granell J, Aguirre-Canyadell M, Nardi J, Tallada N (1984). Malignant fibrous histiocytoma of bone at the site of a total hip arthroplasty. A case report. J Bone Joint Surg.

[23] Weber PC (1986). Epithelioid sarcoma in association with total knee replacement. A case report. J Bone Joint Surg.

[24] Carter RL, Roe FJ (1969). Induction of sarcomas in rats by solid and fragmented polyethylene: experimental observations and clinical implications. Br J Cancer.

[25] Kwon YM, Glyn-Jones S, Simpson DJ, Kamali A, McLardy-Smith P, Gill HS (2010). Analysis of wear of retrieved metal-on-metal hip resurfacing implants revised due to pseudotumours. J Bone Joint Surg.

[26] Mahendra G, Pandit H, Kliskey K, Murray D, Gill HS, Athanasou N (2009). Necrotic and inflammatory changes in metal-on-metal resurfacing hip arthroplasties. Acta Orthop.

[27] Harvie P, Giele H, Fang C, Ansorge O, Ostlere S, Gibbons M (2008). The treatment of femoral neuropathy due to pseudotumour caused by metal-on-metal resurfacing arthroplasty. Hip Int.

[28] Watters TS, Cardona DM, Menon KS, Vinson EN, Bolognesi MP, Dodd LG (2010). Aseptic lymphocyte-dominated vasculitis-associated lesion: a clinicopathologic review of an underrecognized cause of prosthetic failure. Am J Clin Pathol.

[29] De Smet K, De Haan R, Calistri A, Campbell PA, Ebramzadeh E, Pattyn C (2008). Metal ion measurement as a diagnostic tool to identify problems with metal-on-metal hip resurfacing. J Bone Joint Surg Am.

[30] Davies AP, Willert HG, Campbell PA, Learmonth ID, Case CP (2005). An unusual lymphocytic perivascular infiltration in tissues around contemporary metal-on-metal joint replacements. J Bone Joint Surg Am.

[31] Korovessis P, Petsinis G, Repanti M, Repantis T (2006). Metallosis after contemporary metal-on-metal total hip arthroplasty. Five to nine-year follow-up. J Bone Joint Surg Am.

[32] Milosev I, Trebse R, Kovac S, Cor A, Pisot V (2006). Survivorship and retrieval analysis of Sikomet metal-on-metal total hip replacements at a mean of seven years. J Bone Joint Surg Am.

[33] Willert HG, Buchhorn GH, Fayyazi A, Flury R, Windler M, Koster G (2005). Metal-on-metal bearings and hypersensitivity in patients with artificial hip joints. A clinical and histomorphological study. J Bone Joint Surg Am.

[34] Caicedo MS, Desai R, McAllister K, Reddy A, Jacobs JJ, Hallab NJ (2009). Soluble and particulate Co-Cr-Mo alloy implant metals activate the inflammasome danger signaling pathway in human macrophages: a novel mechanism for implant debris reactivity. J Orthop Res.

[35] Campbell P, Shimmin A, Walter L, Solomon M (2008). Metal sensitivity as a cause of groin pain in metal-on-metal hip resurfacing. J Arthroplasty.

[36] Locht H, Skogh T, Kihlstrom E (1999). Anti-lactoferrin antibodies and other types of anti-neutrophil cytoplasmic antibodies (ANCA) in reactive arthritis and ankylosing spondylitis. Clin Exp Immunol.

